# Molecular Detection of *Mycobacterium tuberculosis* in Oral Mucosa from Patients with Presumptive Tuberculosis

**DOI:** 10.3390/jcm9124124

**Published:** 2020-12-21

**Authors:** Barbara Molina-Moya, Nelly Ciobanu, Marta Hernandez, Cristina Prat-Aymerich, Valeriu Crudu, Emily R. Adams, Alexandru Codreanu, Derek J. Sloan, Luis E. Cuevas, Jose Dominguez

**Affiliations:** 1 Institut d’Investigació Germans Trias i Pujol, CIBER Enfermedades Respiratorias (CIBERES), Carretera del Canyet s/n, Camí de les Escoles s/n, Badalona, 08916 Barcelona, Spain; bmolina@igtp.cat (B.M.-M.); cprata@igtp.cat (C.P.-A.); 2 Departament de Genètica i Microbiologia, Universitat Autònoma de Barcelona, Carretera del Canyet s/n Badalona, 08916 Barcelona, Spain; 291097@gmail.com; 3 Institutia Medico-Sanitara Publica, Institutul de Ftiziopneumologie “Chiril Draganiuc”, Strada Constantin Vârnav 13, 2025 Chișinău, Moldova; nelly.ciobanu@gmail.com (N.C.); valeriu.crudu@gmail.com (V.C.); alexandru.codreanu@yandex.com (A.C.); 4 Julius Center for Health Sciences and Primary Care, University Medical Center Utrecht, Utrecht University, Universiteitsweg 100, 3584 CG Utrecht, The Netherlands; 5 Centre for Drugs and Diagnostics, Liverpool School of Tropical Medicine, Pembroke Place, Liverpool L3 5QA, UK; emily.adams@lstmed.ac.uk (E.R.A.); luis.cuevas@lstmed.ac.uk (L.E.C.); 6 School of Medicine, University of St Andrews, North Haugh, St Andrews KY16 9TF, UK; djs26@st-andrews.ac.uk; 7 Department of Clinical Sciences, Liverpool School of Tropical Medicine, Pembroke Place, Liverpool L3 5QA, UK

**Keywords:** oral swab, diagnosis, real-time PCR

## Abstract

Tuberculosis (TB) diagnosis is increasingly based on the detection of *Mycobacterium tuberculosis* complex (MTBC) DNA in sputum using molecular diagnostic tests as the first test for diagnosis. However, sputum can be difficult to obtain in children, patients without productive cough, and the elderly and approaches testing non-sputum samples are needed. We evaluated whether TB can be detected from the oral mucosa of patients with TB. Adults with presumptive TB were examined using culture, Xpert MTB/RIF, smear microscopy and X-Rays. Oral mucosa swabs collected on PrimeStore-MTM, stored at room temperature if tested within 30 days or at −20 °C if examined at a later time. RT-PCR was performed to detect *M. tuberculosis* DNA. Eighty patients had bacteriologically-confirmed TB, 34 had bacteriologically-negative TB (negative tests but abnormal X-rays) and 152 were considered not to have TB (not TB). Oral swabs RT-PCR were positive in 29/80 (36.3%) bacteriologically-confirmed, 9/34 (26.5%) bacteriologically-negative and 29/152 (19.1%) not TB. The yield varied among samples stored for less and more than 30 days (*p* = 0.013) from 61% (11/18) and 29% (18/62) among bacteriologically confirmed, and 30.8% (4/13) and 23.8% (5/21) among bacteriologically-negative participants. Among not TB patients, the specificity was 80.9% (123/152), being 78.3% (18/23) among samples stored less than 30 days and 81.4% (105/129) among samples stored for more than 30 days (*p* = 0.46). The detection of *M. tuberculosis* in oral mucosa samples is feasible, but storage conditions may affect the yield.

## 1. Introduction

Tuberculosis (TB) causes 10 million annual cases and is the leading cause of adult death from an infectious agent [1]. In the last decade, its diagnosis is increasingly based on the detection of *Mycobacterium tuberculosis* complex (MTBC) DNA using molecular diagnostic assays, after the World Health Organization (WHO) recommendation to use these assays as the first test for diagnosis for people with presumptive TB. Most patients are tested using sputum, but these specimens can be difficult to obtain in young children and the elderly, and in patients without productive cough [2].

Recent studies have reported it is possible to recover MTBC DNA from the oral cavity of individuals with bacteriologically confirmed TB [3,4,5]. Although this is an unusual location, MTBC was frequently detected from the tonsils and adenoids of individuals undergoing tonsillectomies in the 19th and 20th centuries, and was proposed as a method to assess the prevalence of TB in the 1930s, and thus the presence of MTBC in the mouth is biologically plausible [6].

In this study, we explored whether patients undergoing routine investigations for TB diagnosis carry MTBC DNA in their oral cavity, to assess its potential as an adjunct test for the diagnosis of TB.

## 2. Materials and Methods

This was a cross sectional study of consecutive adults attending the ambulatory services of the Institute of Phthisiopneumology “Chiril Draganiuc”, Chisinau, Moldova, with a clinical diagnosis of presumptive TB. All patients attending the center were examined with light sputum smear microscopy (Ziehl-Neelsen), liquid culture (BACTEC MGIT 960 TB system, BD, Franklin Lakes, NJ, USA) and X-rays, following the local diagnostic algorithm. X-rays were read by radiologists of the institute and individuals with abnormal X-rays were tested with Xpert MTB/RIF (GX). Participants were classified as bacteriologically-confirmed if the smear-microscopy, GX or culture were positive; bacteriologically-negative TB if the laboratory tests were negative but the X-Rays or/and clinically were considered compatible with TB, and not TB if the laboratory tests were negative and the X-rays were considered to be normal.

Oral mucosa samples were collected before initiation of TB treatment and at the time they attended the laboratory to provide clinical specimens for examination. Samples were collected using flocked swab collection devices (PrimeSwab, Longhorn Vaccines and Diagnostics, San Antonio, TX, USA) by brushing the swab 7–8 times along the inside of the cheek for 10 s. After brushing, the swab was inserted into a tube containing PrimeStore Molecular Transport Medium (PS-MTM), vortexed for 10 s, and left to stand for 30 min at room temperature. According to the manufacturer, specimens can be stored for 30 days at ambient temperature (between 2 °C and 25 °C) or for longer periods if kept frozen between 0 °C and −80 °C. Therefore, as samples were processed at the Institut Germans Trias i Pujol in Spain, samples were stored at room temperature if they would be processed within 30 days, or stored at −20 °C if 30 days passed without having been processed.

DNA was extracted using PrimeXtract total nucleic acid extraction and purification kit (Longhorn Vaccines and Diagnostics, San Antonio, TX, USA). MTBC DNA was detected by real-time polymerase chain reaction (RT-PCR, Longhorn’s PrimeMix MTB Multiplex Amplification Solution) which amplifies the IS6110 and IS1080 regions of the MTBC genome and were conducted using a Lightcycler 480 RT-PCR (Roche, Basel, Switzerland). RT-PCR results were recorded using the endpoint genotyping call of the Lightcycler 480 software. If both “Allele X” and “Allele Y” are called positive, the assay is considered positive. If both alleles are called negative, or if the call is labelled “unknown”, the assay is considered negative. Staff performing the RT-PCR were blinded to the patient information and previous laboratory tests.

We used Chi Square and Fisher’s exact tests to test differences in proportions and Student’s *t*-tests for continuous variables with normal distributions. *p*-values less than 0.05 were considered statistically significant. Analysis was performed using SPSS (SPSS version 15.0, SPSS Inc, Chicago, IL, USA).

All subjects gave their informed consent for inclusion before they participated in the study. The study was conducted in accordance with the Declaration of Helsinki, and the protocol was approved by the Ethics Committee of Liverpool School of Tropical Medicine (18-063), “Chiril Draganiuc” Institute of Phthisiopneumology (8/17 12 June 2017) and Germans Trias i Pujol (PI-16-051).

## 3. Results

A total of 300 participants were enrolled. Of these, 34 (11.3%) were excluded (3 had initiated TB treatment, 12 had extrapulmonary TB, 6 had contaminated culture, and 13 had X-rays with scars considered to be former TB) and 266 were included in the final analysis. The mean (SD) age of the 266 participants was 48.8 (14.4) years and 157 (59.0%) were male. Eighty (30.1%) participants had positive MTBC culture, of which 69 (86.3%) also had positive GX (Table 1), resulting in 80 bacteriologically-confirmed TB. Thirty four (12.8%) participants had negative laboratory tests and abnormal X-rays and were considered bacteriologically-negative TB, while 152 (57.1%) had negative laboratory tests and normal X-rays and were classified as not TB (Table 1). The mean (SD) ages of patients with bacteriologically-confirmed, bacteriologically-negative and not TB were 45.4 (13.0), 40.9 (16.0) and 52.4 (13.7) years, respectively (*p* < 0.001). There was a higher proportion of men and smokers among bacteriologically-confirmed TB participants (Table 1).

Overall, 67 (25.2%) oral swabs were RT-PCR-positive and 199 (74.8%) RT-PCR-negative (Table 2). Fifty-four samples were stored for <30 days and 212 were stored at <30 days and also frozen for a period ranging from 4 and 7 months. Twenty (37.0%) of 54 samples stored for <30 days were RT-PCR positive compared with 47 (22.2%) of the 212 samples also stored for 4–7 months (*p* = 0.03). Among the 80 patients with bacteriologically-confirmed TB, RT-PCR was positive in 11 (61.1%) of 18 samples stored for <30 days and 18 (29.0%) of 62 samples also stored for 4 to 7 months (*p* = 0.01) (Table 2 and Table 3). Among the 34 participants with bacteriologically-negative TB, 4 (30.8%) of 13 samples stored for < 30 days and 5 (23.8%) of 21 samples also stored for 4 to 7 months were RT-PCR-positive (*p* > 0.5). Among patients with not TB, 18 (78.3%) of 23 samples stored for <30 days and 105 (81.4%) of 129 samples also stored for 4–7 months were RT-PCR-negative (*p* = 0.46).

Among bacteriologically-confirmed TB, 20 (43.5%) of 46 smear-positive and 9 (26.5%) of 34 smear-negative patients were RT-PCR-positive (Table 2). Among the 46 smear positives, eight (80.0%) of 10 samples stored for <30 days and 12 (33.3%) of 36 also stored for 4–7 months were RT-PCR-positive, while among the 34 smear-negatives, three (37.5%) of eight samples stored for <30 days (Table 2) and six (23.1%) of 26 samples also stored for 4–7 months were RT-PCR-positive (Table 2). Three (23.3%) of 11 participants with bacteriologically confirmed TB but negative GX and 9 (26.5%) of 34 participants with bacteriologically negative TB had positive RT-PCR.

## 4. Discussion

Our findings demonstrate the feasibility of detecting the presence of MTBC DNA in the oral mucosa of individuals with TB and suggest that testing oral mucosa for TB has potential additional diagnostic value for bacteriological confirmation. Oral swabs are easily collected and are not invasive, and the method would be particularly useful among individuals unable to produce sputum, such as children and people living with HIV. Currently, the method has lower sensitivity that conventional GX and culture and further development is needed to assess whether multiple sampling may increase its sensitivity. Despite the lower sensitivity, we detected MTBC DNA in three individuals with positive culture but negative GX and in patients with a clinical diagnosis but negative laboratory tests and its potential as an adjunct test for diagnosis among patients who do not produce sputum is promising.

A few other studies have assessed the use of oral samples for TB diagnosis. A case-control study of 20 GX-positive participants reported that oral swabs tested using an in-house PCR had 73% sensitivity (87% in smear-positive, 52% in smear-negative) with 100% specificity [3] while a study examining two oral swabs in children with presumptive pulmonary TB obtained 43% sensitivity and 93% specificity among bacteriologically confirmed cases [5]. A further study compared the yield of samples collected from the cheek, tongue and gum [4] and collected single and two consecutive tongue swabs on separate days. The tongue swabs had a higher yield than swabs from other parts of the mouth and two tongue swabs resulted in a combined sensitivity of 83% [4]; suggesting that the sample location, the number of swabs and the time of collection may modify the yield.

Longhorn devices are also suitable to store sputum. Daum et al. reported a sensitivity of 82% (91% specificity) in PS-MTM stored sputum [7]; while Omar et al. reported 100% and 54% sensitivity in smear-positive and smear-negative sputum [8]; and Omar et al. reported a sensitivity of 73% and 85% specificity [9]. As sputum would be expected to have higher bacilli numbers that oral samples, these studies suggest that the lower sensitivity in our study is likely due to lower bacilli numbers.

Our study has limitations that need to be considered. We found that the performance of PS-MTM may be affected by storage conditions. Although the manufacturer indicates DNA can be preserved, if stored frozen, stored samples had a lower yield, and therefore, further prospective studies need to evaluate the time for processing specimens after collection. Patients were also recruited at a reference laboratory, where the pre-test probability of TB was high. Moreover, participants were only screened with GX if the patients had abnormal X-Rays or/and TB clinical symptoms. This procedure may have selected individuals with more advanced TB, who would have been more likely to have cavitations and higher numbers of bacilli. Moreover, we did not collect information on whether participants had gargled with oral disinfectants or if they had consumed foods or drinks before sampling, which may have altered the local amount of bacilli. Altogether, these confounders could have modified the yield of the diagnostic tests. Furthermore, patients were not followed to document the clinical evolution of their illnesses and participants initially classified as not TB could have returned later for further examinations. This latter limitation could explain our lower specificity of the tests compared to other studies. Unfortunately, we did not include healthy individuals for assessing the specificity. However, negative control markers were added in each RT-PCR run and were consistently correct. Finally, although we considered that oral swabs may be useful for children and people with HIV, we did not test these populations, and further studies are needed.

In conclusion, MTBC DNA can be detected from samples collected from the oral cavity in a high proportion of adults with bacteriologically confirmed TB. However, the yield may vary with sample storage conditions. Oral swabbing is a non-invasive procedure and has potential as an adjunct diagnosis for TB and further studies are needed to document its yield in children and individuals with low bacilli load, such as people living with HIV.

## Figures and Tables

**Table 1:** Demographic characteristics of the 266 participants included in the study.

	Bacteriologically Confirmed TB *n* = 80	Bacteriologically Negative TB *n* = 34	Not TB *n* = 152
**Age**			
Mean ± SD * (48.8 ± 14.4)	45.4 ± 13.0	40.9 ± 16.0	52.4 ± 13.7
**Gender**			
Men (157)	60 (75%)	19 (56%)	78 (51%)
Women (109)	20 (25%)	15 (44%)	74 (49%)
**Body mass index**			
<18.5 (36)	9 (11%)	5 (15%)	22 (14%)
18.5–24.9 (122)	33 (41%)	16 (47%)	73 (48%)
25.0–29.9 (77)	26 (33%)	9 (26%)	42 (28%)
>30 (31)	12 (15%)	4 (12%)	15 (10%)
**Smoker**			
No (121)	22 (28%)	19 (56%)	80 (53%)
Yes (145)	58 (73%)	15 (44%)	72 (47%)
**Number of tobacco packs/day**			
1 (101)	42 (72%)	11 (73%)	48 (67%)
2 (31)	10 (17%)	4 (27%)	17 (24%)
3 (13)	6 (10%)	0 (%)	7 (10%)
**Xpert result**			
Positive	69 (86%)	0 (0%)	0 (0%)
Negative	11 (14%)	34 (100%)	0 (0%)
Not performed	0 (%)	0 (0)	152 (100%)

* SD: standard deviation. TB: Tuberculosis

**Table 2:** Oral RT-PCR, for the 54 samples stored at room temperature for less than 30 days, and for the 212 samples stored at −20°C for 4 to 7 months.

	RT-PCR Oral Swabs								
	All			Stored < 30 days			Also Stored for 4 to 7 Months at −20 °C		
	Positive *n* (%)	Negative *n* (%)	Total	Positive *n* (%)	Negative *n* (%)	Total	Positive *n* (%)	Negative *n* (%)	Total
*Bacteriologically confirmed * ^a^	29 (36)	51 (64)	80	11 (61)	7 (39)	18	18 (29)	44 (71)	62
Xpert positive	26 (38)	43 (62)	69	10 (71)	4 (29)	14	16 (29)	39 (71)	55
Smear negative	6 (26)	17 (74)	23	2 (50)	2 (50)	4	4 (21)	15 (79)	19
Smear scanty	6 (26)	17 (74)	23	2 (67)	1 (33)	3	4 (20)	16 (80)	20
Smear 1+	4 (67)	2 (33)	6	-	-	0	4 (67)	2 (33)	6
Smear 2+	3 (75)	1 (25)	4	-	-	0	3 (75)	1 (25)	4
Smear 3+	7 (54)	6 (46)	13	6 (86)	1 (14)	7	1 (17)	5 (83)	6
Xpert negative ^b^	3 (27)	8 (73)	11	1 (25)	3 (75)	4	2 (29)	5 (71)	7
*Bacteriologically-negative * ^c^	9 (27)	25 (74)	34	4 (31)	9 (69)	13	5 (23.8)	16 (76)	21
*Not TB* ^d^	29 (19)	123 (81)	152	5 (22)	18 (78)	23	24 (19)	105 (81)	129
Total	67 (25)	199 (75)	266	20 (37)	34 (63)	54	47 (22)	165 (78)	212

^a^ Positive culture, and variable Xpert and smear. ^b^ Positive culture, negative Xpert and smear. ^c^ Negative culture, Xpert and smear and abnormal X-rays. ^d^ Normal X-Rays and negative culture and smear.

**Table 3:** Sensitivity, specificity, positive predictive value and negative predictive value of oral swab RT-PCR by storage conditions.

	RT-PCR Oral Swabs Performance											
	All Samples				Stored < 30 Days				Also Stored for 4–7 Months at 20 °C			
	Sensitivity (% (*n*) 95%CI)	Specificity (% (*n*) 95%CI)	Positive Predictive Value (% (95%CI))	Negative Predictive Value (% (95%CI))	Sensitivity (% (*n*) 95%CI)	Specificity (% (*n*) 95%CI)	Positive Predictive Value (% (95%CI))	Negative Predictive Value (% (95%CI))	Sensitivity (% (*n*) 95%CI)	Specificity (% (*n*) 95%CI)	Positive Predictive Value (% (95%CI))	Negative Predictive Value (% (95%CI))
Culture	36% (29/80) (26–48)	80% (148/186) (73–85)	43% (31–56)	74% (68–80)	61% (11/18) (36–82)	75% (27/36) (57–87)	55% (32–76)	79% (62–91)	29% (18/62) (19–42)	81% (121/150) (73–86)	38% (25–54)	73% (66–80)
Xpert	40% (26/65) (28–53)	72% (33/46) (56–84)	67% (50–80)	46% (34–58)	71% (10/14) (42–90)	67% (12/18) (41–86)	63% (36–84)	75% (47–92)	29% (16/55) (18–43)	75% (21/28) (55–89)	70% (47–86)	35% (23–48)
TB status ^a^	33% (38/114) (25–43)	81% (123/152) (74–87)	57% (44–69)	62% (55–69)	48% (15/31) (31–67)	78% (18/23) (56–92)	75% (51–90)	53% (35–70)	28% (23/83) (19–39)	81% (105/129) (73–87)	49% (34–64)	64% (56–71)

^a^ Includes bacteriologically-positive and bacteriologically-negative cases. Specificity estimated considering not tuberculosis (TB) cases as not having TB. CI: confidence interval.

## References

[1] World Health Organization (2019). Global Tuberculosis Report. (WHO/CDS/TB/2019.15).

[2] Denkinger C.M., Kik S.V., Cirillo D.M., Casenghi M., Shinnick T., Weyer K., Gilpin C., Boehme C.C., Schito M., Kimerling M. (2015). Defining the needs for next generation assays for tuberculosis. J. Infect. Dis..

[3] Wood R.C., Luabeya A.K., Weigel K.M., Wilbur A.K., Jones-Engel L., Hatherill M., Cangelosi G.A. (2015). Detection of *Mycobacterium tuberculosis* DNA on the oral mucosa of tuberculosis patients. Sci. Rep..

[4] Luabeya A.K., Wood R.C., Shenje J., Filander E., Ontong C., Mabwe S., Africa H., Nguyen F.K., Olson A., Weigel K.M. (2019). Noninvasive Detection of Tuberculosis by Oral Swab Analysis. J. Clin. Microbiol..

[5] Nicol M.P., Wood R.C., Workman L., Prins M., Whitman C., Ghebrekristos Y., Mbhele S., Olson A., Jones-Engel L.E., Zar H.J. (2019). Microbiological diagnosis of pulmonary tuberculosis in children by oral swab polymerase chain reaction. Sci. Rep..

[6] Dwyer-Hemmings L. (2018). ‘A Wicked Operation’? Tonsillectomy in Twentieth-Century Britain. Med. Hist..

[7] Daum L.T., Peters R.P., Fourie P.B., Jonkman K., Worthy S.A., Rodriguez J.D., Ismail N.A., Omar S.V., Fischer G.W. (2015). Molecular detection of *Mycobacterium tuberculosis* from sputum transported in PrimeStore((R)) from rural settings. Int. J. Tuberc. Lung Dis..

[8] Omar S.V., Peters R.P., Ismail N.A., Dreyer A.W., Said H.M., Gwala T., Ismail N., Fourie P.B. (2015). Laboratory evaluation of a specimen transport medium for downstream molecular processing of sputum samples to detect *Mycobacterium tuberculosis*. J. Microbiol. Methods.

[9] Omar S.V., Peters R.P., Ismail N.A., Jonkman K., Dreyer A.W., Said H.M., Gwala T., Ismail N., Fourie P.B. (2016). Field evaluation of a novel preservation medium to transport sputum specimens for molecular detection of *Mycobacterium tuberculosis* in a rural African setting. Trop. Med. Int. Health TM IH.

